# Economic Evaluation of Active Implementation versus Guideline Dissemination for Evidence-Based Care of Acute Low-Back Pain in a General Practice Setting

**DOI:** 10.1371/journal.pone.0075647

**Published:** 2013-10-11

**Authors:** Duncan Mortimer, Simon D. French, Joanne E. McKenzie, Denise A. O′Connor, Sally E. Green

**Affiliations:** 1 Centre for Health Economics, Monash University, Clayton, Victoria, Australia; 2 School of Public Health and Preventive Medicine, Monash University, Melbourne, Victoria, Australia; 3 Centre for Health, Exercise and Sports Medicine, The University of Melbourne, Melbourne, Victoria, Australia; University of Louisville, United States of America

## Abstract

**Introduction:**

The development and publication of clinical practice guidelines for acute low-back pain has resulted in evidence-based recommendations that have the potential to improve the quality and safety of care for acute low-back pain. Development and dissemination of guidelines may not, however, be sufficient to produce improvements in clinical practice; further investment in active implementation of guideline recommendations may be required. Further research is required to quantify the trade-off between the additional upfront cost of active implementation of guideline recommendations for low-back pain and any resulting improvements in clinical practice.

**Methods:**

Cost-effectiveness analysis alongside the IMPLEMENT trial from a health sector perspective to compare active implementation of guideline recommendations via the IMPLEMENT intervention (plus standard dissemination) against standard dissemination alone.

**Results:**

The base-case analysis suggests that delivery of the IMPLEMENT intervention dominates standard dissemination (less costly and more effective), yielding savings of $135 per x-ray referral avoided (-$462.93/3.43). However, confidence intervals around point estimates for the primary outcome suggest that – irrespective of willingness to pay (WTP) – we cannot be at least 95% confident that the IMPLEMENT intervention differs in value from standard dissemination.

**Conclusions:**

Our findings demonstrate that moving beyond development and dissemination to active implementation entails a significant additional upfront investment that may not be offset by health gains and/or reductions in health service utilization of sufficient magnitude to render active implementation cost-effective.

## Introduction

Clinical practice has proved remarkably resilient to recommendations for practice change embedded in clinical practice guidelines (CPGs) [1]. While it seems clear that simply developing and disseminating CPGs will not necessarily produce improvements in clinical practice, further evidence is required to justify moving beyond development and dissemination to active implementation [2], [3]. Over the past decade, various strategies for the active implementation of CPGs for acute low back pain (LBP) have been trialled in general practice [4]–[10] and allied health settings [11]–[13]. While several studies demonstrate that active implementation of CPGs for acute LBP can improve GP practice and patient health outcomes [5], [10], it should be remembered that active implementation typically entails an upfront investment that may not be fully offset by health gains or reductions in health service utilization.

Evidence regarding the incremental costs(savings) of active implementation of CGPs for LBP – as well as its effects on clinical practice and health outcomes – is available from one study conducted in an allied health setting [12] and one study conducted in a general practice setting [14]. Based on comparison between active implementation plus standard dissemination against standard dissemination of a CPG [15] in a sample of 113 Dutch physiotherapists, Hoeijenbos et al. [12] concluded that “it is very likely that the extended implementation strategy incurs extra costs without producing health gains, hence it is very likely to be not cost-effective” (p93). In the only other available study, Becker et al. [14] compared (i) multifaceted implementation of a CPG [16], (ii) motivational counselling plus multifaceted implementation, and (iii) standard dissemination in a sample of 1322 LBP patients from 76 GP practices. Becker et al. [14] concluded that a trend towards cost-effectiveness is visible in their data but suggest that this trend should be confirmed in future studies.

The IMPLEMENT trial aimed to test the effectiveness and cost-effectiveness of a multifaceted and theory-informed intervention: the IMPLEMENT intervention, for implementing a CPG [17] for acute LBP in general medical practice in Victoria, Australia. The control intervention was standard dissemination. This article reports findings from the cost-effectiveness analysis. Results from the effectiveness analyses are reported in full elsewhere [18]. We hypothesised that:

- H1. After taking account of reductions in health service use, the IMPLEMENT intervention will be less costly than standard dissemination.
- H2. The IMPLEMENT intervention will increase GP adherence to key messages of the CPG (providing advice on activity/bed rest and referral for any imaging).
- H3. The IMPLEMENT intervention will dominate (less costly but of at least equivalent effect) standard dissemination.

## Methods

### Ethics

Ethical approval for this study was obtained from the Monash University Standing Committee on Ethics in Research involving Humans (2006/047). All participants provided written informed consent.

### Study design

The IMPLEMENT study [ACTRN012606000098538] is a cluster randomized controlled trial (CRT), with the clusters being general practices of one or more GPs drawn from a sampling frame of 1688 general practices within the state of Victoria, Australia. Ninety-two practices where at least one general practitioner (GP) agreed to participate were randomized to either a control group or an intervention group. For each included practice, other GPs in the same practice were invited to participate and given the opportunity to object to the practice participating. Included GPs were offered professional development points and access to LBP experts. Planned analyses described in the trial protocol [19] required each included practice to enrol an average of 25 patients (2300 patients). Cost-effectiveness analyses were conducted alongside the IMPLEMENT study to quantify the additional costs (savings) and health gains associated with the implementation strategy as compared with standard dissemination from a health sector perspective. The time period for inclusion of relevant costs and consequences was set at 12 months after delivery, consistent with the timing of outcome measurement in the trial (described below).

### Control

Practices randomized to the control group received access to the CPG for acute LBP as per the standard dissemination strategy [17]. The standard dissemination strategy comprised development of user-friendly material for the target audiences (clinicians and consumers), a range of methods to access the information, publicising the availability of the materials, endorsement by professional and lay associations and approval by Australia's National Health and Medical Research Council (NHMRC). All documents were and are available electronically via the NHMRC website [20]. In addition, the summary (user-friendly) version of the guideline for clinicians, which includes the consumer information sheets, was distributed by post to approximately 40,000 GPs and other clinicians across Australia. While the control intervention closely approximates the standard dissemination strategy described above, a printed copy of the guideline and a written reminder of how to access the electronic version of the CPG were sent to control group practices shortly after randomization.

### Intervention

Practices randomized to the intervention arm received active implementation of the CPG for acute LBP. A novel multifaceted and theory-based implementation strategy, tailored to the general practice context, was developed to overcome modifiable barriers and encourage enablers for implementation of two key messages specific to the LBP CPG. These key messages are that (i) diagnostic x-rays are rarely necessary in the management of acute LBP and (ii) that remaining active reduces pain and disability. Development of a strategy to implement these key messages was undertaken as described elsewhere [21]. The resulting multifaceted and theory-based implementation strategy – the IMPLEMENT intervention – consisted of a facilitated interactive workshop run over two sessions each of three hours duration, distribution of an intervention pack to workshop participants, and postal distribution of a DVD to GPs in the intervention group. The two workshop sessions employed multiple behaviour change techniques including reflection on the GP participant's own management of patients with acute LBP, small group discussion, persuasive communication, modelling, rehearsal, scripting and action planning. Each workshop session was directed by a trained GP facilitator supported by two of the study investigators (co-facilitators). Session I (*Confidence in diagnosis*) focused on x-ray referral and included videotaped role play with a peer expert and live role play with actors trained to simulate patient presentation and to role play x-ray seeking behaviour. Session II (*Move it or lose it*) focused on advice to stay active and included role play in participant pairs using pre-prepared scripts and creation/adaptation of scripts for use in GP participant's own clinical practice. Detailed schedules of activity for each workshop session have been reported elsewhere [21].

### Outcomes

The planned analyses described in the trial protocol relied on patient-level outcomes to measure the effects of the intervention on health-related quality of life, pain-related disability, physical function and physical pain [18]. Unfortunately, these patient-level measures could not be collected due to failure of patient recruitment; necessitating a departure from planned analyses [22]. The cost-effectiveness analyses described here instead rely on intermediate outcome measures reflecting GP adherence to, or departure from, the key messages embedded in the CPG as summarised in Table 1.

**Table 1:** Schedule of measures for economic evaluation.

Measure	Data collection instrument	Timing	Source	Level at which data are collected
X-ray occurred^1,2^; CT scan occurred 3; X-ray or CT scan occurred^4^	Medicare data	12 months	Medicare Australia	GP
Advice to stay active; Advised bed rest; X-ray referral; Any imaging referral	Questionnaire (patient vignettes)	12 months	GP	GP
Direct costs of developing intervention	Data abstraction;	On completion of development	Admin records	Intervention
	Interview		Project officers	
Direct costs of delivering intervention	Data abstraction;	On completion of delivery to all GPs	Admin records	Intervention
	Interview		Project officers	

1 Primary outcome.

2 Medicare data: number of referrals for all lumbar spine and pelvis x-ray services by each included GP for a 12 month period after the intervention/control was delivered.

3 Medicare data: number of referrals for all lumbar spine and pelvis CT scan services by each included GP for a 12 month period after the intervention/control was delivered.

4 Medicare data: number of referrals for all lumbar spine and pelvis x-ray or CT scan services by each included GP for a 12 month period after the intervention/control was delivered.

Observations on behaviours consistent with the CPG *from actual clinical practice* were limited to the number of lumbar spine and pelvis x-ray referrals for any condition by each GP during the 12 months after delivery of intervention/control. The number of x-ray referrals is therefore taken as the primary outcome for the economic evaluation. Observations from actual clinical practice were not available for ‘advice to stay active’ or ‘advice to take bed rest’. Observations on each of the key behaviours consistent with the CPG (imaging referral, advice to stay active and advice to take bed rest) at 12 months after delivery of intervention/control were, however, available in *simulated* clinical practice. Specifically, GP adherence in simulated practice for each of the key clinical behaviours was measured by GP responses to a series of four acute LBP patient vignettes. We conducted supplementary analyses for adherence to the guideline in simulated practice as measured by these vignettes.

In order to evaluate whether any increase in adherence to the CPG has come at a reasonable price, we also measured differences in cost between the treatment and control groups. Variation with respect to cost within the trial period derives from (i) development of the IMPLEMENT intervention, (ii) delivery of the IMPLEMENT intervention, (iii) delivery of the control intervention, and (iv) any subsequent changes in practice and subsequent health effects. A summary of unit costs by category of resource use is provided in Appendix S1. Data sources for (i), (ii), (iii) and (iv) are detailed in Appendices S2 to S5 respectively. Total costs for each individual provider were calculated by summing together provider-level data with respect to health service utilization, total development cost per provider and total delivery cost per provider.

### Analyses

Methods for calculating development and delivery costs for the treatment group are described in Appendices S2 and S3, respectively. Methods for calculating delivery costs for the control group are described in Appendix S4. Methods for calculating imaging costs per GP for the treatment and control groups are described in Appendix S5.

We estimated the effect of the intervention by running separate regressions for each outcome (cost and adherence). We regressed each outcome on to a dummy variable designating randomization to the treatment or control group, as well as controls for design strata and pre-specified confounders at the GP and practice level (including GP age, years since GP graduated, self-reported special interest in LBP, number of GPs per practice, practice method of billing, rural/metro practice). Given the structure of our data and the advice of Buntin and Zaslavsky [23], we used Generalised Estimating Equations (GEEs), with robust variance estimation (sandwich variance estimator), to account for the correlation of responses within practices and used the method of recycled predictions to obtain incremental effects [24].

To inform the decision of whether or not to proceed with roll out of an existing intervention, we calculated the ratio of undiscounted incremental costs (excluding development costs) and benefits. To inform the decision of whether to invest in development and subsequent delivery of an intervention, we calculate the ratio of discounted incremental costs and outcomes. Methods for discounting are described in Appendix S6. Results from the economic evaluation were expressed as: (i) additional costs (savings) per x-ray referral avoided and (ii) additional costs (savings) per additional consultation adherent to the key messages of the CPG in simulated practice.

Point estimates for incremental cost-effectiveness were calculated as the average incremental effect of treatment status on total cost per GP divided by the average incremental effect of treatment status on the relevant outcome. Confidence intervals around the incremental cost-effectiveness ratio were derived by application of Fieller's Theorem using iprogs.do [24]. Cost-effectiveness acceptability curves (CEACs) were used to visualise uncertainty associated with the decision to replace the control intervention with the evaluated intervention.

## Results

### Participants

The study sample comprised 112 GPs drawn from the 92 included practices. Forty-five practices (59 GPs) were randomized to the intervention group and 47 to the control group (53 GPs). Of the 59 GPs randomized to the intervention group, 54 participated in the trial per protocol and received the IMPLEMENT intervention (either in person or via the DVD recording). Baseline characteristics of practices and GPs are reported elsewhere [18].

### Adherence to the CPG

Data were available for imaging referral, design strata and all pre-specified potential confounders for 84 of the 112 GPs in the intention to treat (ITT) sample. Table 2 reports adjusted and unadjusted rates of imaging referral per GP for GPs with complete data. Results are also reported for discounted referrals (see Appendix S6). Adjusted incidence rate ratios suggest that intervention group GPs referred for x-ray at a rate 0.83 times lower than control group GPs (p = 0.211). Incremental effects reported in Table 2 suggest that exposure to the intervention reduced x-ray referrals by −3.43 (95%CI: −9.45, 2.59; p = 0.260) but this reduction did not reach statistical significance.

**Table 2:** Effect of the intervention on imaging referral, with and without discounting.

Variable	No. practices (no. GPs)		Mean (SD)		Adj IRR^1,2^ (95%CI)	Incremental Effect^3^ (SE)^4^
	Rx	Control	Rx	Control		
X-ray	34 (44)	37 (40)	14.6 (12.1)	19.2 (14.6)	0.83 (0.61, 1.12)	−3.43 (3.10)
X-ray @ 5%discount	34 (44)	37 (40)	13.9 (11.5)	18.3 (13.9)	0.82 (0.61, 1.11)	−3.27 (2.95)
X-ray @ 3%discount	34 (44)	37 (40)	14.2 (11.7)	18.6 (14.1)	0.83 (0.61, 1.11)	−3.33 (3.01)
X-ray @ 7%discount	34 (44)	37 (40)	13.7 (11.3)	17.9 (13.6)	0.82 (0.61, 1.11)	−3.21 (2.89)

1 Adjusted rate ratios estimated from models fitted using xtgee, family(nbinomial “estimated heterogeneity parameter”) link(log) vce(robust) exposure(total Medicare patients) where intercept derived from nbreg. All models adjusted for the following design strata and pre-specified confounders: GP age (years), years since GP graduated, self-reported special interest in LBP, number of GPs per practice, practice method of billing, rural/metro practice.

2 IRR  =  incidence rate ratio. Estimate of intervention effect adjusted for design strata and potential confounders (specified prior to undertaking the analysis).

3 Incremental effect  =  change in referral per GP due to exposure to the intervention after controlling for design strata and pre-specified potential confounders. Here, incremental effects derived from model predicted values using method of recycled predictions [24].

4 Standard errors derived from bootstrap using bsmultiv.do [24].

Table 3 reports adjusted and unadjusted rates of adherence to the key messages of the CPG in simulated practice. Adjusted odds ratios reported in Table 3 suggest that, after controlling for design strata and pre-specified confounders, adherence in simulated practice was statistically significantly more common in the intervention group than in the control group with respect to x-ray (OR = 1.76, p = 0.045), imaging (OR = 2.36, p<0.001) and activity (OR = 4.49, p = 0.001). Non-adherence to the CPG with respect to bed-rest was very uncommon in both treatment and control groups; resulting in very large standard errors and large magnitude but statistically insignificant treatment effects (OR = 2.91, p = 0.354). Incremental effects reported in Table 3 suggest that exposure to the intervention increased the probability of adherence by 0.099 (95%CI: −0.002, 0.201; p = 0.056) in the case of x-ray; by 0.177 (95%CI: 0.068, 0.286; p = 0.002) in the case of imaging; and 0.297 (95%CI: 0.210, 0.384; p = 0.000) in the case of activity. Applying these probabilities to 25^th^ percentile estimates of the number of LBP patients seen per year (52 patients/year) for GPs enrolled in the trial [18] gives a projected treatment effect of 5.2 additional patients treated as per the guideline per GP per year in the case of x-ray adherence, of 9.2 additional patients treated as per the guideline per GP per year in the case of imaging adherence, and 15.4 additional patients per GP per year in the case of activity adherence.

**Table 3:** Effect of the intervention on adherence as measured by the vignettes.

Variable	No. practices (no. GPs)		Rx group adherence		Control group adherence		Adj OR^1^ (95%CI)	Incremental Effect^2^ (SE)^3^
	Rx	Control	No.	%	No.	%		
**X-ray** 4	31 (38)	36 (40)	126/152	(83)	109/160	(68)	1.76* (1.01, 3.05)	0.099 (0.052)
**Imaging** 4	31 (38)	36 (40)	119/152	(78)	89/160	(56)	2.36**(1.48, 3.79)	0.177**(0.056)
**Activity** 5	31 (38)	36 (40)	121/152	(80)	82/160	(51)	4.49**(1.90,10.60)	0.297**(0.044)
**Bed rest** 6	34 (41)	38 (43)	163/164	(99)	168/171	(98)	2.91 (0.30,27.83)	0.011 (0.012)

* : p<0.05; **: p<0.01.

**X-ray adherence** defined as GPs not referring for a lumbosacral plain x-ray.

**Imaging adherence** for vignettes was defined as GPs not referring for any of following three diagnostic tests: lumbosacral plain x-ray, lumbar CT scan, lumbar MRI.

**Activity adherence** defined as “Advise the patient to continue with their normal daily activities” regardless of other interventions selected (“Paracetamol”, “Non-steroidal anti-inflammatory drugs”, “Advise the patient to do specific back exercises”, “Advise the patient to do general exercises (e.g. walking)”,”Manual therapy”, “Referral to another health care provider”, “Other”).

**Bed rest adherence** defined as either not recommending “Bed rest”, or recommending “Bed rest” for ≤ 2 days.

1 Adjusted Odds Ratio (OR)  =  Estimate of intervention effect adjusted for design strata and potential confounders (specified prior to undertaking the analysis). Adjusted OR estimated from models fitted using xtgee family(binomial) link(logit) vce(robust) yielding semi-robust standard errors.

2 Incremental effect  =  change in probability that simulated consult will be adherent to the key messages of the CPG due to exposure to the intervention after controlling for design strata and potential confounders (specified prior to undertaking the analysis). Here, incremental effects derived from model predicted values using method of recycled predictions [24].

3 Standard errors derived from bootstrap using bsmultiv.do [24].

4 Models adjusted for the following design strata and pre-specified potential confounders: GP age (years), years since GP graduated, self-reported special interest in LBP, number of GPs per practice, practice method of billing, rural/metro practice.

5 Models adjusted for the following design strata and pre-specified potential confounders: GP age (years), years since GP graduated, self-reported special interest in LBP, number of GPs per practice, practice method of billing, rural/metro practice, baseline measure of fear-avoidance beliefs.

6 No adjustment for stratification variables or potential confounders because of limited events of non-adherence.

### Cost of development and delivery

The cost of intervention development for the IMPLEMENT intervention was calculated at $83,455.79. In the base case, this amount was amortized under the assumption that the IMPLEMENT intervention will eventually be delivered to the entire cohort of Australian GPs but will bear no repeated or wider use. We estimate some 39,000 GPs to be practising in Australia at the time of roll out, based on Australian Government Data [25]. Assuming a participation rate of 2.6% of the target population, which was the recruitment rate for the IMPLEMENT trial, the cost of intervention development is then spread over some 1014 GPs. Under this approach, the per participant cost of development is estimated at $82.30 per GP participant. This assumption was varied in sensitivity analysis firstly to include full development costs (under the assumption that the IMPLEMENT intervention is specific to the study population and bears no repeated use) and secondly to exclude all development costs (recognizing the potential for repeated application of the strategy in other settings and for other indications). Table 4 reports total cost for treatment and control groups for the base-case, as well as with full and zero development costs. Table 4 also reports results for discounted total costs (see Appendix S6).

**Table 4:** Effect of the intervention on total cost, with and without discounting.

Variable	No. practices (no. GPs)		Mean (SD)		Exp. Coef.^1^ (95%CI)	Incremental Cost^2^ (SE)^3^
	Rx	Control	Rx	Control		
Base-case^4^	34 (44)	37 (40)	$4612 (3239)	$4941 (3208)	0.92 (0.70, 1.22)	−$375.55 (724)
No dev cost^4^	34 (44)	37 (40)	$4529 (3239)	$4941 (3208)	0.91 (0.69, 1.20)	−$462.93 (723)
Full dev cost^6^	34 (44)	37 (40)	$5944 (3239)	$4941 (3208)	1.21 (0.95, 1.54)	+$1023.26 (695)
Base-case^4^ @5%	34 (44)	37 (40)	$4396 (3085)	$4705 (3055)	0.93 (0.70, 1.22)	−$353.50 (690)
Base-case^4^ @3%	34 (44)	37 (40)	$4480 (3145)	$4797 (3115)	0.92 (0.70, 1.22)	−$362.06 (703)
Base-case^4^ @7%	34 (44)	37 (40)	$4315 (3027)	$4617 (2998)	0.93 (0.70, 1.22)	−$345.27 (676)
No dev cost^5^ @5%	34 (44)	37 (40)	$4313 (3085)	$4705 (3055)	0.91 (0.69, 1.20)	−$440.89 (688)
Full dev cost^6^ @5%	34 (44)	37 (40)	$5728 (3085)	$4705 (3055)	1.22 (0.96, 1.56)	+$1044.69 (661)

1 Exponentiated coefficients and incremental effects estimated from models fitted using xtgee, family(gamma) link(log) vce(robust) yielding semi-robust standard errors. All models adjusted for the following design strata and pre-specified confounders: GP age (years), years since GP graduated, self-reported special interest in LBP, number of GPs per practice, practice method of billing, rural/metro practice.

2 Incremental cost  =  change in total cost per GP due to exposure to the intervention after controlling for design strata and pre-specified potential confounders. Here, incremental cost derived from GEE predicted values using method of recycled predictions [24].

3 Standard errors derived from bootstrap using bsmultiv.do [24].

4 Development (amortized), delivery and imaging cost.

5 Delivery and imaging cost only. Cost of development for the IMPLEMENT intervention excluded.

6 Development (full), delivery and imaging cost.

Delivery costs for the IMPLEMENT intervention were attributed to individual GPs based on individual-level records of attendance at workshop sessions, receipt of the DVD, and self-reported viewing of the DVD. For example, the cost of workshop delivery averaged over the 36 intervention group GPs who attended both workshops (none attended just one of the workshops) was calculated at $9743.87/36 = $270.66 per workshop attendee. The cost of DVD distribution averaged over the 49 intervention group GPs remaining in the trial at the time of distribution was calculated at $2454.30/49 = $50.09 per DVD distributed. The cost of delivering the IMPLEMENT intervention excluding workshop delivery, DVD distribution and DVD-based self-education averaged over the 59 GPs randomized to the intervention group was calculated at $15,091.40/59 = $255.79 per intervention group GP. The cost of delivering the control intervention averaged over the 53 GPs randomized to the control group was calculated at $6,453.30/53 = $121.76 per control group GP. After attributing direct intervention costs to individual GPs, total cost per GP participant for treatment and control groups was obtained as the summation of development, delivery and imaging costs per GP participant in treatment and control groups, respectively.

For the base-case analysis (with development costs amortized over Australian GPs), total cost per GP was lower for the intervention group than for the control group and this remained the case after adjusting for design strata and pre-specified confounders. Adjusted incidence rate ratios reported in Table 4 suggest that total cost per GP was 0.92 times lower in the intervention group than control group GPs (p = 0.578). Incremental effects reported in Table 4 suggest that exposure to the intervention reduced total cost per GP by $375.55 (95%CI: −$1815.63, $1064.53; p = 0.605) but this reduction in total cost did not reach statistical significance.

### Cost effectiveness of delivery

**Figure 1:**
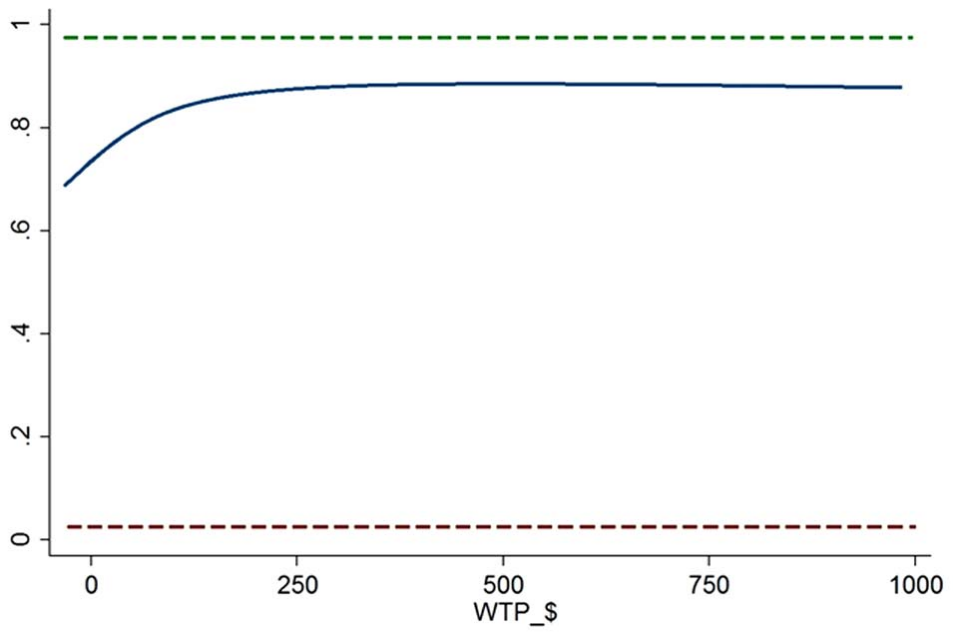
Cost effectiveness acceptability curve for x-ray referral.

To inform the decision of whether or not to proceed with delivery of IMPLEMENT (*ex post* of development), we calculated the ratio of undiscounted incremental costs (excluding development costs) and undiscounted incremental benefits. The base-case analysis suggests that delivery of the IMPLEMENT intervention dominates standard dissemination (less costly and more effective), yielding savings of $135 per x-ray referral avoided (−$462.93/3.43). However, confidence intervals around point estimates suggest that – irrespective of willingness to pay – we cannot be at least 95% confident that the IMPLEMENT intervention offers better value than standard dissemination. Figure 1 gives the cost-effectiveness acceptability curve (CEAC) for the primary outcome. The CEAC (solid blue line) plots the relative frequency, or probability, that the IMPLEMENT intervention is cost-effective, compared to standard dissemination at varying levels of willingness to pay (WTP) per x-ray referral avoided. While the acceptability curve never intersects the upper limit of the 95% confidence interval (dashed green line), 80% of the density is cost-effective at a WTP of around $100 per x-ray or imaging referral avoided.

Incremental cost effectiveness ratios for adherence in simulated practice were expressed as cost per additional consult adherent to the key messages of the CPG per 52 consults (rather than as cost per point improvement in the probability of adherence). The base-case analysis suggests that delivery of the IMPLEMENT intervention is less costly and more effective than standard dissemination; yielding savings of $89 per additional consult adherent for x-ray (−$462.93/5.2), savings of $50 per additional consult adherent for imaging (−$462.93/9.2), and savings of $30 per additional consult adherent for advice to stay active (−$462.93/15.4). Confidence intervals around these point estimates suggest that – given a sufficiently high willingness to pay for the outcome of interest and assuming that increased probability of adherence in simulated practice is predictive of increased probability of adherence in actual practice of a sufficient scale – we can be at least 95% confident that development and delivery of the IMPLEMENT intervention represents good value in comparison to standard dissemination. If fund-holders are willing to pay at least $458 per additional consult adherent for x-ray, then delivery of the IMPLEMENT intervention represents good value in comparison to standard dissemination. Thresholds for other outcomes were $123 and $66 per additional consult adherent for imaging and advice to say active, respectively.

The threshold level of willingness to pay (WTP) was highly sensitive to assumptions regarding the extent to which increased adherence in simulated practice translates into increased adherence in actual practice. If we were to assume that an increased probability of adherence occurred in just 10 consults per GP, then the threshold WTP becomes $2342 per additional consult adherent for x-ray. At the limit, if increased adherence in simulated practice fails to translate into increased adherence in actual practice, then there is no WTP at which we can be 95% confident that the intervention with the larger point estimate for effect represents good value compared with the alternative.

### Cost effectiveness of development and delivery

To inform the decision of whether to invest in development and subsequent delivery, we calculated the ratio of discounted incremental costs and discounted incremental adherence. Results from this analysis will be most useful to policy-makers and clinicians considering undertaking development and subsequent delivery of an active implementation strategy (*ex ante* of development). The base-case analysis suggests that development and delivery of the IMPLEMENT intervention dominates standard dissemination; yielding savings of $108 per discounted x-ray referral avoided (−$353.50/3.27). Confidence intervals around point estimates suggest that – irrespective of willingness to pay – we cannot be at least 95% confident that the IMPLEMENT intervention differs in value from standard dissemination. For decision-makers interested in lower levels of confidence, over 80% of the density is cost-effective if WTP exceeds $100 per discounted x-ray referral avoided.

Incremental cost effectiveness ratios for adherence in simulated practice were expressed as discounted cost per discounted consult adherent to the key messages of the CPG per 52 consults. The base-case analysis suggests that delivery of the IMPLEMENT intervention is less costly and more effective than standard dissemination; yielding savings of $72 per additional discounted consult adherent for x-ray (−$353.50/4.9), savings of $40 per additional consult adherent for imaging (−$353.50/8.8), and savings of $24 per additional discounted consult adherent for activity (−$353.50/14.7). Confidence intervals around the point estimate for x-ray adherence suggest that – irrespective of willingness to pay – we cannot be at least 95% confident that the IMPLEMENT intervention differs in value from standard dissemination. For decision-makers interested in lower levels of confidence, over 80% of the density is cost-effective if WTP exceeds $50 per additional discounted consult adherent for x-ray referral.

### Sensitivity analysis

Results (not reported here but available upon request) confirmed that findings were qualitatively identical under the majority of alternative assumptions considered in sensitivity analysis. The inclusion of full intervention development costs did, however, fundamentally alter our findings. When full development costs were included, development and delivery of the IMPLEMENT intervention no longer dominated standard dissemination. Under this scenario, the IMPLEMENT intervention was more costly and more effective than standard dissemination at an additional cost of $313 per (5%) discounted x-ray referral avoided (+$1023.26/3.27). Confidence intervals around point estimates suggest that – irrespective of willingness to pay – we cannot be at least 95% confident that the IMPLEMENT intervention offers better value than standard dissemination.

## Discussion

The cost-effectiveness analysis described here relies on intermediate outcomes reflecting adherence to, or departure from, the key messages embedded in the CPG. Specifically, that remaining active reduces pain and disability and that diagnostic x-rays yield additional health risks but only a small chance of additional health benefits in the management of acute LBP [20]. With regards to the possible health benefits of diagnostic x-rays, one recent Australian study found that less than 1% of patients presenting to general practice with acute LBP were later confirmed as having a spinal fracture; the majority of which were identifiable from the application of red-flags on initial presentation [26]. With regards to the additional health risks of diagnostic x-rays, recent evidence estimates excess cancer mortality from radiation exposure at approximately one death per 25,000 lumbar spine and pelvis x-ray examinations [27], [28].

Results reported in this paper suggest that, for the primary outcome, there is no willingness to pay at which we can be at least 95% confident that the IMPLEMENT intervention offers better value than standard dissemination. Decision-makers may, however, make decisions at lower levels of confidence. For the cost-effectiveness of delivery, over 80% of the density is cost-effective at a WTP of around $100 per x-ray referral avoided. Decision-makers must then consider whether $100 is a reasonable price to pay per x-ray avoided. For a threshold WTP of $100 per x-ray avoided, we can estimate the implicit WTP per cancer death avoided by multiplying out over excess cancer mortality risks from radiation exposure. With excess cancer mortality from radiation exposure of one death per 25,000 lumbar spine and pelvis x-ray examinations, decision-makers accepting a threshold WTP of $100 per x-ray are implicitly accepting a threshold WTP of $2,500,000 per excess cancer death avoided.

In interpreting our results, several limitations of our analysis should be borne in mind. First, the present study estimates the relative cost-effectiveness of the intervention as implemented and may not reflect the relative cost-effectiveness under a wider roll out. Note, for example, that GPs were not paid an honorarium for their attendance at workshops (though they did receive continuing medical education (CME) points for their participation). While those GPs who participated without monetary compensation clearly felt that the improvement in their practice and attendant CME points were sufficient compensation for their time, many GPs might only be willing to participate for a fee and some might be unwilling to participate at any price. Our base-case analysis assumes a participation rate of 2.6% of the current cohort of Australian GPs in any wider roll out. It is likely that participation rates will differ from context to context due, for example, to stronger or weaker incentives for CME (versus additional throughput or leisure) under fee-for-service versus capitated payment. Where higher or lower participation rates are expected in a particular context, our ‘full development’ or ‘no development’ scenarios may be more pertinent to that context. Along similar lines, the cost effectiveness of the IMPLEMENT intervention in a particular context will vary according to the incentives for imaging referral, the existence of capacity constraints or rationing for imaging services, and the prior or parallel implementation of other interventions for guideline adherence.

Treatment effects were estimated in GPs for whom data on the relevant outcome/cost, design strata and all pre-specified potential confounders were available. In the case of imaging referral, data was available for the relevant outcome (imaging referral), design strata and all pre-specified potential confounders for 84 of the 112 GPs in the ITT sample. Treatment effects conditioned on design strata only were also estimated for the 90 GPs in whom data was available on imaging referrals and design strata (but not pre-specified confounders). Results from these analyses are available upon request but were consistent with the results presented here.

Finally, our analysis has been conducted from a health sector perspective. Any benefits to the GP or to the patient arising from non-adherence to the key messages of the CPG (i.e. referral contra to the guidelines may nonetheless be a source of non-health utility in the form of reassurance to patients or fear-avoidance to GPs) have therefore been excluded from the analysis. On the cost side of the equation, our analysis excludes direct and indirect costs outside the health sector (including waiting time and travel time to attend treatment, any productivity gains due to a change in specific disability, and time lost from work associated with treatment), as well as certain indirect health care costs associated with between-group variation in health status (including use of over-the-counter or prescription analgesics, allied health or GP consults, and the time of volunteer or paid carers). While the omission of indirect health care costs represents a departure from planned analyses, such plans were predicated on access to patient self-report data on service utilization and patient-level outcomes that were not forthcoming due to failed patient recruitment. No attempt has been made to extrapolate from intermediate outcomes (adherence to the guideline in actual or simulated practice or behavioural intention) to variation in health status and thence to variation in productivity and service utilization.

## Conclusion

Our findings demonstrate that, after taking account of reductions in service use, adopting a more active approach to implementation via the IMPLEMENT intervention may actually be less costly than standard dissemination. Unfortunately, this outcome is far from certain and there remains a good chance that wider roll out of the IMPLEMENT intervention would impose additional costs upon the health system. While this result does not exclude the possibility that the IMPLEMENT intervention is cost effective, an argument must be made that any additional cost is a cost worth paying. Making such an argument based on the results reported here would require us to assume: that behavioural simulations are predictive of clinical practice and/or that society places a high value on improvements in clinical practice and the consequent health gains. We conclude that our results are not, by themselves, supportive of a wider roll out of the IMPLEMENT intervention.

## Supporting Information

Appendix S1 **Unit costs by category of resource use.**
(DOCX)

Appendix S2 **Cost analysis for development of the active intervention strategy.**
(DOCX)

Appendix S3 **Delivery of the active implementation strategy.**
(DOCX)

Appendix S4 **Cost analysis for delivery of the control intervention.**
(DOCX)

Appendix S5 **Practice change & subsequent health effects.**
(DOCX)

Appendix S6 **Adjustment for differential timing of costs and effects.**
(DOCX)
