# Supplementary material for: Economic Evaluation of Active Implementation versus Guideline Dissemination for Evidence-Based Care of Acute Low-Back Pain in a General Practice Setting
Source: PLoS One. 2013 Oct 11;8(10):e75647. doi: 10.1371/journal.pone.0075647 (PMC3795707; doi:10.1371/journal.pone.0075647)
Supplement: Appendix S1 — Unit costs by category of resource use. (DOCX) [file pone.0075647.s001.docx]

**Appendix S1:** **Unit costs by category of resource use**

Unit costs for health service resource use (see Table S1) were as per the Manual of Resource Items for use in submissions to the Commonwealth of Australia’s Pharmaceutical Benefits Advisory Committee (PBAC). Resource use of marketed goods and services outside the health sector and not included in the Manual was valued at market prices. Unmarketed goods and services were valued at their opportunity cost.

**Table S1: Unit costs by category of resource use**

| Category | **Description** | **Unit cost** | **Source** |
| --- | --- | --- | --- |
| **Overheads** | | | |
| Office space | Rental charge per year per m^2^ for office-space and utilities on the Monash Medical Centre Campus. | $233.68 | 2009 Monash University direct facilities charges |
| **Personnel** | | | |
| Investigator time | Level B, Step 1 salary of $86,352 plus 35.14% salary on-costs | $116,696 | 2009 Monash Academic Salary Scale |
| Project Officer | Level A, Step 3 salary of $64,355 plus 35.14% salary on-costs | $86,969 | 2009 Monash Academic Salary Scale |
| Admin Officer | HEW 4, Step 4 salary of $46,906 plus 35.14% salary on-costs | $63,389 | 2009 Monash Professional Staff Salary Scale |
| Simulated patients | Hourly rate of simulated patient time inflated to March 2009 AUD: Cost*CPI_03/09 / CPI_06/07=$23.69*(166.2/157.5) | $25.00 | Administrative records of costs incurred |
| Peer expert | Basic lecture (2 hours preparation/training time per 1 hour of delivery) | $137.14 | 2008/09 sessional academic teaching rates at Monash University |
| Initial session: focus group facilitator | Basic lecture (2 hours preparation/training time per 1 hour of delivery) | $137.14 | 2008/09 sessional academic teaching rates at Monash University |
| Repeat session: focus group facilitator | Repeat lecture (1 hour preparation per 1 hour delivery). | $91.43 | 2008/09 sessional academic teaching rates at Monash University |
| DVD filming & editing | Hourly out-of-hours rate for Monash Multimedia staff | $112.50 | Administrative records of costs incurred |
| GP attendance at focus groups | Honorarium paid in 2007 AUD inflated to March 2009 AUD: Honorarium* CPI_03/09 / CPI_06/07=$200*(166.2/157.5) | $211.05 | Administrative records of costs incurred |
| GP attendance / self-education time | Workshops were conducted on weekends or after-hours and so we cost attendance at the opportunity cost of lost leisure time. | $0.00 | Drummond et al (1997) suggest a value for lost leisure time *of zero in the base case* but concede that arguments could also be made for valuing lost leisure time at the average wage rate or at average overtime rates. See Drummond M, O’Brien B, Stoddart G, Torrance G (1997) Methods for the economic evaluation of health programmes. New York: OUP. |
| Travel time | Per hour travel time cost for non-CBD car travel during peak period in 2003 AUD inflated to March 2009 AUD: Cost* CPI_03/09 / CPI_06/03=$8.00*(166.2/133.8) | $9.94 | Booz, Allen, Hamilton (2003) ACT Transport demand elasticities study. Canberra: Commonwealth Department of Urban Services. |
| **Venue hire & catering** | | | |
| Monash Conference Cntr: Midweek | High-quality seminar room with a 15 person capacity (cluster set-up) per four hour half-day. | $272.00 | Monash University Conference Centre Hire Rates |
| Monash Conference Cntr: Weekend | High-quality seminar room with a 15 person capacity (cluster set-up) per hour. | $110.00 | Monash University Conference Centre Hire Rates |
| Meeting Room: Half-day or Evening | High-quality training rooms with a 25-30 person capacity in suburban / rural Victoria per half-day or evening session. | $150.00 | PivotWest (formerly known as Western Melbourne DGP) |
| Meeting catering | Per person per half-day charge for coffee / tea and biscuits. | $4.80 | Monash University Conference Centre Hire Rates |
| Workshop catering | Per person per 3 hour session for deluxe lunch package. | $23.50 | Monash University Conference Centre Hire Rates |
| **Other consumables** | | | |
| Direct transport costs incurred 2007 | Direct costs of taxi and private car travel to metro and rural focus groups inflated to March 2009 AUD: Cost*CPI_03/09 / CPI_06/07=$454.00*(166.2/157.5) | $479.08 | Administrative records of costs incurred |
| Direct accom. costs incurred 2007 | Direct costs of accommodation prior to rural focus groups inflated to March 2009 AUD: Cost*CPI_03/09 / CPI_06/07=$572.00*(166.2/157.5) | $603.60 | Administrative records of costs incurred |
| Purchase of AMPCo database | Purchase cost of AMPCo database inflated to March 2009 AUD: Cost*CPI_03/09 / CPI_06/07=$1,155.00*(166.2/157.5) | $1,218.80 | Administrative records of costs incurred |
| CMHSE Direct admin charge | CMHSE charge per hour for administration / coordination of simulated patients inflated to March 2009 AUD: Cost*CPI_03/09 / CPI_06/07=$50.00*(166.2/157.5) | $52.76 | Administrative records of costs incurred |
| Transcription | Transcription was completed by an external consultant at a cost of $2,970.00 for 22 hours of recorded material, implying a unit cost of $135 for transcription of each hour of recorded material. | $135.00 | Administrative records of costs incurred |
| In-house printing | 2009 advertised retail prices for black and white printing at Officeworks | $0.08 | Office Works Price List |
|  | 2009 advertised retail prices for colour printing at Officeworks | $1.08 |  |
| Professional printing | Run of 65 x 2 in prescription pad format (100pp) inflated to March 2009 AUD: Cost*CPI_03/09 / CPI_06/07=$2,646.69*(166.2/157.5) | $2792.89 | Administrative records of costs incurred |
| DVD production | Per unit cost of materials, burning and packaging for DVDs | $2.50 | Administrative records of costs incurred |
| Advertising | Half page (120 x 190mm) advertisement in monochrome or spot blue | $250.00 | [Adelaide](http://www.awdgp.org.au) West General Practice Network |
| Phone calls | Untimed local phone calls | $0.30 | Telstra Price List |
| DL postage | Australia Post postage effective 15 September 2008 for within-Australia delivery of DL envelope | $0.55 | Australia Post Price List |
| C5 postage | Australia Post postage effective 15 September 2008 for within-Australia delivery of large letter C5 envelope | $1.10 |  |
| DVD postage | Australia Post postage effective 15 September 2008 for within-Australia delivery of prepaid Parcel Post Satchel | $5.70 |  |
| **Health Service Utilization** | | | |
| X-ray | MBS Item 57715 - Radiology: Pelvic Girdle (R) | $60.90 | Schedule of Medicare Benefits (2009) |
|  | MBS Item 58106 - Radiology: Spine Lumbosacral (R) | $77.00 | Schedule of Medicare Benefits (2009) |
|  | MBS Item 58108 - Radiology: Spine, four regions, cervical, thoracic, lumbosacral and sacrococcygeal (R) | $132.90 | Schedule of Medicare Benefits (2009) |
|  | MBS Item 58109 - Radiology: Spine Sacrococcygeal (r) | $47.00 | Schedule of Medicare Benefits (2009) |
|  | MBS Item 58112 - Radiology: Spine, two examinations of the kind referred to in items 58100, 58103, 58106 and 58109 (R) | $97.25 | Schedule of Medicare Benefits (2009) |
|  | MBS Item 58115 - Radiology: Spine, three examinations of the kind mentioned in items 58100, 58103, 58106 and 58109 (R) | $132.90 | Schedule of Medicare Benefits (2009) |
| CT scan | MBS Item 56223 - Computed Tomography: scan of spine, lumbosacral region, without intravenous contrast medium, payable once only, whether 1 or more attendances are required to complete the service (R) (K) (Anaes.) | $240.00 | Schedule of Medicare Benefits (2009) |
|  | MBS Item 56226 - Computed Tomography: scan of spine, lumbosacral region, with intravenous contrast medium and with any scans of the lumbosacral region of the spine prior to intravenous contrast injection when undertaken; only 1 benefit payable whether 1 or more attendances are required to complete the service (R) (K) (Anaes.) | $351.40 | Schedule of Medicare Benefits (2009) |
|  | MBS Item 56233 - Computed Tomography: scan of spine, two examinations of the kind referred to in items 56220, 56221 and 56223 without intravenous contrast medium payable once only, whether 1 or more attendances are required to complete the service (R) (K) (Anaes.) | $240.00 | Schedule of Medicare Benefits (2009) |
|  | MBS Item 56234 - Computed Tomography: scan of spine, two examinations of the kind referred to in items 56224, 56225 and 56226 with intravenous contrast medium and with any scans of these regions of the spine prior to intravenous contrast injection when undertaken; only 1 benefit payable whether 1 or more attendances are required to complete the service (R) (K) (Anaes.) | $351.40 | Schedule of Medicare Benefits (2009) |
|  | MBS Item 56237 - Computed Tomography: scan of spine, three regions cervical, thoracic and lumbosacral, without intravenous contrast medium, payable once only, whether 1 or more attendances are required to complete the service (R) (K) (Anaes.) | $240.00 | Schedule of Medicare Benefits (2009) |
