# Supplementary material for: Economic Evaluation of Active Implementation versus Guideline Dissemination for Evidence-Based Care of Acute Low-Back Pain in a General Practice Setting
Source: PLoS One. 2013 Oct 11;8(10):e75647. doi: 10.1371/journal.pone.0075647 (PMC3795707; doi:10.1371/journal.pone.0075647)
Supplement: Appendix S2 — Cost analysis for development of the active intervention strategy. (DOCX) [file pone.0075647.s002.docx]

**Appendix S2: Cost analysis for development of the active intervention strategy**

Resource use associated with the development of the IMPLEMENT intervention was costed based on financial and administrative records and a detailed description of the development process obtained from the project manager and project officers. Administrative and financial records provided data as to the number of focus group informants, total person hours spent in focus groups and interviews for informants and facilitators, use and location of interview and meeting rooms, total person time for data analysis and interpretation of findings, membership of the advisory committee and total person hours for advisory committee members.

Table S2 summarises the resource-based costing for development of the IMPLEMENT intervention. Resource use for development of the IMPLEMENT intervention can be divided into a number of categories:

***Administration:*** Resource-use associated with administration and coordination of intervention development can be attributed to personnel, overheads including office space, and consumables.

- *Personnel:* The development of the IMPLEMENT intervention was co-ordinated over a period of 12 months by the IMPLEMENT Project Officer, with assistance from the IMPLEMENT Administrative Officer, with duties including recruitment of GP informants; arranging meeting / training rooms for focus groups; liaising with venues, facilitators, co-facilitators and informants regarding availability and arrangements for focus groups; production and distribution of materials to GP informants; and participation in other aspects of the intervention development process. While both the IMPLEMENT Project and Administration Officers were employed full-time for the full 12 month period, much of this time was spent on tasks associated with research / evaluation that were excluded from the cost-analysis. Based on administrative records regarding timing of intervention development and of competing research / evaluation tasks, we estimate 0.4 fraction of the IMPLEMENT Project Officer’s time and 0.2 of the IMPLEMENT Admin Officer’s time was devoted to development of the IMPLEMENT intervention.
- *Overheads:* Use of office space and utilities were estimated using the Monash University internal cost recovery formula for central services; based on the total time required for administration / coordination of intervention delivery (48 weeks from a 48 week year), the fraction of this time period spent on administration / coordination duties for the IMPLEMENT Project (0.4) and Admin Officers during this time (0.2 EFT), and the maximum allowance in square metres for staff with the same level and function as the IMPLEMENT Project and Admin Officers (6m^2^ for Academic Staff Level A plus 6m^2^ for supporting Administrative Staff).
  - *Consumables:* Calls for GP informants were placed in local Divisions of General Practice (DGP) newsletters. Consumables relating to administration and coordination include telephone calls to DGP to arrange placement of advertising in DGP newsletters, production / distribution of advertising, and production/distribution of two-page confirmation letters to GP informants.

***GP Advisory Group Meetings:***

- - *Personnel:* An eleven member GP advisory committee was recruited from university research institutions, divisions of general practice, primary care practice, and the Royal Australian College of GPs. The advisory committee provided advice regarding the intervention development process and intervention design. The advisory committee attended one hour meetings with five of the IMPLEMENT investigators on two occasions during development of the intervention.
  - *Venue hire:* Advisory group meetings were held at the MIHSR offices, with meeting room hire and catering provided by the University.

***Focus groups:*** A series of focus group interviews was run with GP informants to identify barriers and enablers to implementation of key messages from the CPG.

- - *Personnel:* Each focus group was led by a trained facilitator supported by the IMPLEMENT Project Officer. Delivery of the focus groups and preparation time for the facilitator only are included as basic (2 hours preparation/training time per 1 hour of delivery) and repeat (1 hour preparation per 1 hour delivery) lectures for initial and repeat delivery. Pro-rated salary for the IMPLEMENT Project Officer has been included under administrative costs and no further allowance has been made for his role in supporting focus group sessions.
  - *Venue hire:* Focus group meetings were held at the MIHSR offices, with meeting room hire and catering provided by the University.
  - *Consumables:* A two-page information sheet summarising the broad questions to be asked during focus group sessions was distributed to GP informants with their confirmation letter. Information sheets were printed in-house in black and white.
  - *GP informant time costs:* The 43 GP informants recruited for the intervention development phase were split across 11 focus groups, each of 2 hours duration. Each focus group was led by a trained facilitator supported by the IMPLEMENT Project Officer (SF). We assume a mean 30 minutes travel time to and from the venue for each of the attending GP informants.

***Analysis and interpretation of focus group data:***

- - *Personnel:* Focus group sessions were recorded for later transcription. Transcription of 22 hours of recorded material was completed by an external consultant. Transcripts were analysed and interpreted by the IMPLEMENT Project Officer (SF) and one of the study investigators (DO’C). Analysis and interpretation of each focus group transcript entailed approximately 16 person hours, split roughly equally across the IMPLEMENT Project Officer and the study investigator. After excluding the time of the IMPLEMENT Project Officer (SF) to avoid double-counting, investigator time attributable to analysis and interpretation ran to 88 person hours (8 hours per transcript over 11 transcripts).

***Intervention Design:*** The tailored implementation strategy was designed by study investigators (DO’C, JF, JG, JMc, NS, PS, RB, SF, SG, SM) based the barriers/enablers identified from focus group data, consideration of the available evidence, and the expert opinion of study investigators. Study investigators met on thirteen occasions for a total duration of 28 hours and 126 person hours. Attendance at intervention design meetings averaged five investigators but varied from a minimum of three to a maximum of eight.

- - *Personnel:* After excluding the time of the IMPLEMENT Project Officer (SF) to avoid double-counting, investigator time attributable to intervention development ran to 98 person hours.
  - *Venue hire:* Advisory group meetings were held at the MIHSR offices, with meeting room hire and catering provided by the University.

***Formulating the patient scenarios:*** Patient scenarios were formulated to emphasise barriers and enablers for the management of acute LBP consistent with the CPG. Patient scenarios were formulated by the IMPLEMENT Project Officer (SF) and SG over a period of one week using MIHSR office space.

- - *Personnel:* Pro-rated salary plus salary on-costs of 35.14% for one week of a 48 week year has been included to cover SG’s participation in formulating patient scenarios. Pro-rated salary for the IMPLEMENT Project Officer has been included under administrative costs and no further allowance has been made for SF’s participation in formulating the patient scenarios so as to avoid double-counting.
  - *Venue hire:* MIHSR office space for the IMPLEMENT Project Officer has been captured under overheads for administration/coordination of intervention development.

**Table 6: Summary of resource-based costing for development of the IMPLEMENT intervention**

| **Input** | **Number (A)** | **Unit cost (B)** | **Total cost (A x B)** | |
| --- | --- | --- | --- | --- |
| Administration / coordination | | | | |
| IMPLEMENT Project Officer | Level A, Step 3 at 0.4 FTE over 12 months | $64,355 plus 35.14% salary on-costs=  $86,969 | $34,787.60 | |
| IMPLEMENT Administration Officer | HEW 4, Step 4 at 0.2 FTE over 12 months | $46,906 plus 35.14% salary on-costs = $63,389 | $12,677.80 | |
| Office space | 6m^2^ for 0.4 EFT and 6m^2^ for 0.2 EFT over 12 months (6x0.4)+(6x0.2)=3.6m^2^) | $233.68/m^2^ | $841.25 | |
| Liaison with venues, facilitators / informants | 86 untimed local phone calls | $0.30 | $25.80 | |
| Recruitment of GP Informants | | | | |
| Phone calls to DPGs | 30 | $0.30 | $9.00 | |
| Half-page monochrome advert in DGP Newsletters | 5 | $250.00/advert | $1250.00 | |
| Confirmation letters | 43 confirmation letters @ 1 page each = 43pp | $0.08 | $3.44 | |
| Postage | Within-Australia postage for 43 DL confirmation letters | $0.55 | $23.65 | |
| GP Advisory Group Meeting | | | | |
| Attendance | 16 persons x 2 sessions x 60mins = 32hrs | $0.00 | $0.00 | |
| Travel time | 16 persons x 2 sessions x 60mins = 32hrs | $9.94 | $318.08 | |
| Venue hire | 2 sessions | $150.00 | $300.00 | |
| Meeting catering | 16 persons x 2 sessions = 32 person sessions | $4.80 | $153.60 | |
| Focus Groups | | | | |
| RF facilitator | 1 initial sessions x 2 hrs per session = 2 hrs | $137.14 / hr | $274.28 | |
|  | 10 repeat sessions x 2 hrs per session = 20 hrs | $91.43 / hr | $1,828.60 | |
| IMPLEMENT Project Officer | 1 initial sessions x 2 hrs per session = 2 hrs | $64,355 plus 35.14% salary on-costs= $86,969 | Included under administrative costs | |
|  | 10 repeat sessions x 2 hrs per session = 20 hrs |  |  |  |
| GP attendance | 43 honorariums | $211.05 | $9,075.15 | |
| Travel time | 43 GPs x 60mins = 43 hrs | $9.94 | $427.24 | |
| Direct travel costs | Transport to metro and rural focus groups for RF facilitator and IMPLEMENT Project Officer | $479.08 | $479.08 | |
|  | Accommodation before rural focus group for RF facilitator and IMPLEMENT Project Officer | $603.60 | $603.60 | |
| Venue hire | 11 focus group sessions | $150.00 | $1,650.00 | |
| Meeting catering | 43 GP sessions plus 22 facilitator/project officer sessions = 55 person sessions | $4.80 | $264.00 | |
| Materials | 43 information sheets @ 2pp each=86pp | $0.08 | $6.88 | |
| Analysis & interpretation | | | | |
| Transcription | 11 focus groups x 2 hrs = 22 hrs recorded material | $135 / hr | $2,970.00 | |
| RF investigator time | 11 focus groups x 8 hrs = 88 hrs ≅ 0.046 FTE (2.20*40 hr wk in 48 wk yr) | $86,352 plus 35.14% salary on-costs= $116,696 | $5,368.02 | |
| IMPLEMENT Project Officer | 11 focus groups x 8 hrs = 88 hrs | $64,355 plus 35.14% salary on-costs= $86,969 | Included under administrative costs | |
| Intervention Design | | | |  |
| RF investigator time | 98 person hours ≅ 0.05 FTE (2.45*40 hr wk in 48 wk yr) | $86,352 plus 35.14% salary on-costs= $116,696 | $5,834.80 | |
| IMPLEMENT Project Officer | 28 person hours | $64,355 plus 35.14% salary on-costs= $86,969 | Included under administrative costs | |
| Venue hire | 13 meetings | $150.00 / session | $1,950.00 | |
| Patient Scenarios | | | |  |
| RF Investigator time | 1 wk of 48 wk yr ≅ 0.02 FTE | $86,352 plus 35.14% salary on-costs= $116,696 | $2,333.92 | |
| IMPLEMENT Project Officer | 1 wk of 48 wk yr ≅ 0.02 FTE | $64,355 plus 35.14% salary on-costs= $86,969 | Included under administrative costs | |
| Venue hire | 1 wk of office space | $233.68/m^2^ | Included as overheads under admin costs | |
| **Total** |  |  | **$83,455.79** | |
