# Supplementary material for: Economic Evaluation of Active Implementation versus Guideline Dissemination for Evidence-Based Care of Acute Low-Back Pain in a General Practice Setting
Source: PLoS One. 2013 Oct 11;8(10):e75647. doi: 10.1371/journal.pone.0075647 (PMC3795707; doi:10.1371/journal.pone.0075647)
Supplement: Appendix S3 — Delivery of the active implementation strategy. (DOCX) [file pone.0075647.s003.docx]

**Appendix S3: Delivery of the active implementation strategy**

In addition to access to the CPG via the existing dissemination strategy, the intervention group received the IMPLEMENT intervention; a multifaceted intervention specifically designed to address the barriers and enablers for implementation of key messages from the CPG. The intervention consisted of a facilitated interactive workshop run over two sessions each of three hours duration, distribution of an intervention pack to workshop participants, and postal distribution of a DVD to GPs in the intervention group. Each workshop session was directed by a trained GP facilitator supported by two of the study investigators (co-facilitators). Detailed schedules of activity for each workshop session have been reported elsewhere.[21] Workshop participants were provided with an intervention pack comprising all supporting materials for the workshop plus patient education materials for use in participants’ routine practice (education handouts and a patient activity log in a prescription pad format). The DVD included selected film footage from Sessions I and II of the workshop plus electronic resources regarding evidence-based management of acute LBP. The video footage on the DVD included the didactic material and did not include the small group discussions. The total DVD running time was 180 minutes.

Fifty-nine GPs were randomized to the intervention group, 54 of whom received the IMPLEMENT intervention (either in person or via the DVD recording). Cost items associated with delivery of the IMPLEMENT intervention included costs associated with administering and coordinating delivery of the intervention; production of materials for workshops including the intervention pack; venue hire and catering; opportunity cost of GP time spent in travel, workshop and self-education; and labour costs associated with preparation/delivery/facilitation of workshops.

Resource use associated with the delivery of the IMPLEMENT intervention was estimated from administrative and financial records detailing resource use associated with the production of materials, total person hours spent in organising and facilitating workshops, total hours of GP attendance, venue location and total hours venue hire. Any costs associated with development and standard dissemination of the CPG under existing practice were assumed to arise in equal magnitude for intervention and control groups and were excluded from further consideration. Table S3 summarises the resource-based costing for delivery of the IMPLEMENT intervention. Resource use and total costs for relevant cost categories were calculated as follows:

***Administration:*** Resource-use associated with administration and coordination of the IMPLEMENT intervention can be attributed to personnel, overheads including office space, and consumables.

- *Personnel:* The delivery of the IMPLEMENT intervention was co-ordinated over a period of six weeks by the IMPLEMENT Project Officer, with assistance from the IMPLEMENT Administrative Officer, with duties including recruitment of GP facilitators and GP participants; arranging meeting / training rooms for workshop sessions; liaising with venues, facilitators, co-facilitators and participants regarding availability and arrangements for workshops sessions; production of materials including intervention packs and DVDs; mail distribution of DVDs. While both the IMPLEMENT Project and Administration Officers were employed full-time over a longer period than this six week period, much of the project and administration officers’ time was spent in development of the IMPLEMENT intervention (described above) and on tasks associated with research / evaluation (and therefore excluded from the cost-analysis). Based on administrative records regarding timing of intervention delivery and of competing research / evaluation tasks, we estimate a 0.6 fraction over 6 weeks was devoted to administrating and coordinating delivery of the IMPLEMENT intervention.
- *Overheads:* Costs associated with office space and utilities were estimated using the Monash University internal cost recovery formula for central services; based on the total time required for administration / coordination of intervention delivery (6 weeks from a 48 week year), the fraction of this time period spent on administration / coordination duties for the IMPLEMENT Project and Admin Officers during this time (0.6 fraction), and the maximum allowance in square metres for staff with the same level and function as the IMPLEMENT Project and Admin Officers (6m^2^ for Academic Staff Level A plus 6m^2^ for supporting Administrative Staff).
- *Consumables:* Consumables relating to administration and coordination include telephone calls to venues, facilitators and participants and postage / printing associated with inviting GP participants to attend. Recruitment entailed a mass mail-out of invitations to every GP resident in Victoria (4996 GPs in 1688 general practices) and included in the AMPCo database, yielding a recruitment rate across both arms of the trial of 2.6%. We attribute half of this cost of GP recruitment to the intervention group.

***Facilitator Training & Workshops:***

- - *Personnel:* Workshop sessions were generally facilitated by one of five GPs who were each experienced in teaching medicine at either an undergraduate or post-graduate level and who had each received specific training in the content and aims of the workshop. We ran Session I on five occasions and Session II on six occasions. For seven of the nine sessions, the GP facilitator was assisted by two of the study investigators (co-facilitators). However, for two of the full-day workshops (4 sessions), none of the GP facilitators were available and the two co-facilitators delivered these sessions. Facilitator training entailed provision of materials (the intervention guide plus a schedule of behaviour change techniques to be delivered) plus telephone discussion with one of the study investigators covering the content and aims of the workshop. Delivery and preparation time (including facilitator training time) of the two-day course are included as basic (2 hours preparation/training time per 1 hour of delivery) and repeat (1 hour preparation per 1 hour delivery) lectures for initial and repeat delivery. While facilitator training time has been captured in the unit cost per hour of delivery, personnel costs associated with facilitator training (ie, telephone discussion with study investigators) have not and so we allow an additional hour investigator time per GP facilitator. The peer expert attended the first workshop session for one hour, with subsequent sessions shown DVD footage of the role play performed at this first session. The initial delivery and preparation time for the peer expert role play were costed as a basic (2 hours preparation/training time per 1 hour of delivery) lecture. Simulated patients were obtained from the Simulated Patient Program at Monash University’s Centre for Medical and Health Sciences Education (CMHSE). The number of simulated patients required and available for each session varied between one and six, depending upon the number of attending GPs and the location and timing of each session. Across all sessions, the IMPLEMENT intervention entailed 24 person hours of simulated patient time. We assume a mean 30 minutes travel time to and from each workshop session for all workshop personnel.
  - *Venue hire:* Workshops were held at MIHSR (2 evening sessions, 2 week-end sessions), Monash Conference Centre (3 evening sessions, 2 week-end sessions), and University of Melbourne School of Rural Health (2 week-end sessions). Workshops were generally split across two weekday evenings (5 evening sessions) but both Session I and II were held as an all day Saturday workshop on three occasions (3 full days). Attendance at workshop sessions varied between two and fourteen GPs plus (1) facilitators and (2) co-facilitators. We make the conservative assumption that commercial rates would be paid for venue hire and catering for replication or wider roll out of the IMPLEMENT intervention.
  - *Consumables:* An intervention guide (40 pp) plus patient education handouts in prescription pad format (50pp) and a patient activity log in a prescription pad format (50pp) were provided to each GP who attended the workshop. Each GP facilitator received the intervention pack (140pp in total) together with a schedule of behaviour change techniques to be delivered (29 pp). Materials in prescription pad format were printed professionally as a run of 130 (65x2) pads. Intervention guides and the schedule of behaviour change techniques were printed in-house, predominantly in black and white but four pages of each intervention guide distributed to GPs were printed in colour. We make the conservative assumption that commercial rates would be paid for production of materials in any replication or wider roll out of the IMPLEMENT intervention. Development costs for materials included in the intervention pack and for the schedule of behaviour change techniques were captured in costings for *development* of the IMPLEMENT intervention.
  - *GP time costs:* Of the 59 GPs randomized to the intervention group, 36 GPs attended both Session I and Session II of the six hour workshop (three hours per session). While the majority of workshop sessions were run from venues in Melbourne, only eight of the 36 GPs who attended the workshops practiced in a rural location and a further proportion of those practicing in rural Victoria were able to attend workshop sessions at the University of Melbourne’s School of Rural Health located in central Victoria. We assume a mean 30 minutes travel time to and from each workshop session for each of the attending GPs.

***Self-education:***

- - *GP time costs:* Fourteen GPs reported that they had viewed the DVD (of whom eight had also attended the workshop and six had not); implying a total time commitment of 42 hours for completion of the DVD component of the intervention.
  - *Consumables:* The IMPLEMENT intervention included distribution of a DVD to all GPs in the intervention group. The Monash University Video Production Unit completed production, post production, duplication and packaging of the DVD, entailing 9 hours total filming time (including set-up time) across two workshop sessions plus an estimated 9 hours editing/post-production. Sixty DVDs were pressed and packaged, with one DVD distributed by post to each of the 49 intervention group GPs who had not dropped out of the trial at the time of distribution.

**Table S3: Summary of resource-based costing for delivery of the IMPLEMENT intervention**

| **Input** | **Number (A)** | **Unit cost (B)** | **Total cost (A x B)** |
| --- | --- | --- | --- |
| Administration / coordination | | | |
| IMPLEMENT Project Officer | Level A, Step 3 at 0.6 over 6 weeks (0.6x6/48=0.075 EFT) | $64,355 plus 35.14% on costs=  $86,969 | $6,522.68 |
| Administration Officer | HEW 4, Step 4 at 0.6 over 6 weeks (0.6x6/48=0.075 EFT) | $46,906 plus 35.14% on-costs = $63,389 | $4,754.18 |
| Office space | 12m^2^ for 0.6 over 6 weeks (12x0.6x6/48=0.9m^2^) | $233.68/m^2^ | $210.31 |
| CMHSE admin charges^ | 4 hrs admin/coordination of simulated patients | $52.76 | $211.05 |
| Liaison with facilitators, venues & participants | 72 untimed local phone calls | $0.30 | $21.60 |
| Recruitment of GP participants | | | |
| Purchase of AMPCo database | 1 | $1,218.80 | $1,218.80 |
| Mail out | 2498 invitation to participate letters @ 2pp each = 4996pp | $0.08 | $399.68 |
| Postage | Within-Australia postage for 2498 DL invitation letters | $0.55 | $1,373.90 |
| Facilitator training | | | |
| Investigator time | 5 x 1 hr = 0.003 FTE (0.125*40 hr wk in a 48 wk yr) | $86,352 plus 35.14% on-costs= $116,696 | $350.10 |
| Facilitator time | Included as preparation time for each workshop session | - | - |
| Phone calls | 5 untimed local phone calls | 0.30 | $1.50 |
| Intervention (Rx) Guides | 5 Rx guides @ 40pp each plus 5 schedules @ 29pp each=345 pp | $0.08 / page | $27.60 |
| Patient education handouts | Run of 65 x 2 in prescription pad format (100pp) | Included in costing for two-day workshop | |
| Two-day workshop | | | |
| GP facilitators | 5 initial sessions x 3 hrs per session = 15 hrs | $137.14 / hr | $2,057.10 |
|  | 2 repeat sessions x 3 hrs per session = 6 hrs | $91.43 / hr | $548.58 |
| RF co-facilitators | (2 initial sessions x 2 persons) x 3 hrs / person session = 12 hrs | $137.14 / hr | $1,645.68 |
|  | (7 repeat sessions x 2 persons) x 3 hrs / person session = 42 hrs | $91.43 / hr | $3,840.06 |
| Peer expert & DVD production | Peer expert (1 session x 60mins) =1 hr | $137.14 / hr | $137.14 |
|  | DVD production | Included under DVD production costs for GP self-education DVDs | |
| Simulated patients | 24 hrs | $25.00/ hr | $600.00 |
| Travel time | Peer expert (1 session x 60mins) =1 hr; 20 simulated patients x 60mins = 20 hrs; GP facilitators (7 sessions x 60 mins) = 7 hrs; RF co-facilitators (11 sessions x 2 persons x 60 mins) = 22 hrs; 36 GPs x 2 sessions x 60mins per session= 72 hrs | $9.94 | $1212.68 |
| GP attendance time | 36 GPs x 2 sessions x 3 hrs per session= 216 hrs | $0.00 | $0.00 |
| Venue hire | Monash CC: 2 week-end sessions @ 3 hrs per session | $110.00 / hr | $660.00 |
|  | Monash CC: 2 evening sessions | $272.00 / session | $816.00 |
|  | MIHSR / UniMelb: 6 sessions | $150.00 / session | $900.00 |
| Workshop catering | (36 GPs x 2 sessions) plus 29 facilitator/co-facilitator sessions = 101 person sessions | $23.50 per person session | $2,373.50 |
| Intervention guides, b&w printing | 54 intervention guides @ 36pp each in b&w = 1944 pp | $0.08 / page | $155.52 |
| Intervention guides, colour printing | 54 intervention guides @ 4pp each in colour = 216 pp | $1.08 / page | $233.28 |
| Patient education handouts | Run of 65 x 2 in prescription pad format (100pp) | $2792.89 | $2792.89 |
| Self-education | | | |
| DVD viewing time | 14 GPs x 3 hrs viewing time = 42 hrs | $0.00 | $0.00 |
| DVD production & distribution | 18 hours total filming & editing/post-production time for DVDs | $112.50 | $2025.00 |
|  | DVD production including packaging for 60 DVDs | $2.50 | $150.00 |
|  | Postage & packaging for 49 DVDs | $5.70 | $279.30 |
| **Total** |  |  | **$35,518.13** |

^CMHSE charge for administration / coordination of simulated patients.
