# Supplementary material for: Economic Evaluation of Active Implementation versus Guideline Dissemination for Evidence-Based Care of Acute Low-Back Pain in a General Practice Setting
Source: PLoS One. 2013 Oct 11;8(10):e75647. doi: 10.1371/journal.pone.0075647 (PMC3795707; doi:10.1371/journal.pone.0075647)
Supplement: Appendix S4 — Cost analysis for delivery of the control intervention. (DOCX) [file pone.0075647.s004.docx]

**Appendix S4: Cost analysis for delivery of the control intervention**

Practices randomized to the control group received access as per the standard dissemination strategy plus a written reminder of the key messages from the CPG. Costs associated with standard dissemination of the CPG are common and invariant to both treatment and control groups and, on this basis, have not been explicitly included in the cost analysis reported here. While the control intervention closely approximates standard dissemination, a colour printed copy of the full guideline (covering LBP plus four other conditions and totaling 85pp in A5 format) and a written reminder of how to access the electronic version of the CPG was sent to 53 GPs in the 47 control group practices at the same time that the IMPLEMENT intervention was delivered to intervention group GPs. While copies of the guideline were provided to IMPLEMENT by the developer at no charge, we assume that full commercial rates would be paid for production of materials in any replication or wider roll out of the control intervention. Resource use associated with the development of the IMPLEMENT intervention was costed based on financial and administrative records and a detailed description of the development process obtained from the project manager and project officers. Administrative and financial records provided data as to the number of focus group informants, total person hours spent in focus groups and interviews for informants and facilitators, use and location of interview and meeting rooms, total person time for data analysis and interpretation of findings, membership of the advisory committee and total person hours for advisory committee members.

Resource use associated with the delivery of control intervention was estimated from administrative and financial records detailing resource use associated with the sending written reminders to control group practices detailing how to access the CPG. Any costs associated with development and standard dissemination of the CPG under existing practice were assumed to arise in equal magnitude for intervention and control groups and were excluded from further consideration. Table S4 summarises the resource-based costing for delivery of the control intervention.

**Table S4: Summary of resource-based costing for delivery of the control intervention**

| **Input** | | **Number (A)** | **Unit cost (B)** | **Total cost (A x B)** |
| --- | --- | --- | --- | --- |
| Postal dissemination of the CPG | | | | |
|  | IMPLEMENT Administration Officer | HEW 4, Step 4 at 0.2 over 1 week (0.2/48=0.004 EFT) | $46,906 plus 35.14% on-costs = $63,389 | $253.56 |
|  | Distribution list | Purchase of AMPCo database | $1,218.80 | $1,218.80 |
|  | Printing | 86pp per GP for 53 GPs = 4558pp | $1.08 | $4,922.64 |
|  | Postage | Within-Australia postage for 53 C5 envelopes | $1.10 | $58.30 |
|  | GP self-education time | 20minutes per GP for 53 GPs=17.50hrs | $0.00 | $0.00 |
| **Total** | |  |  | **$6,453.30** |
