# Supplementary material for: Economic Evaluation of Active Implementation versus Guideline Dissemination for Evidence-Based Care of Acute Low-Back Pain in a General Practice Setting
Source: PLoS One. 2013 Oct 11;8(10):e75647. doi: 10.1371/journal.pone.0075647 (PMC3795707; doi:10.1371/journal.pone.0075647)
Supplement: Appendix S5 — Practice change & subsequent health effects. (DOCX) [file pone.0075647.s005.docx]

**Appendix S5: Practice change & subsequent health effects**

Cost items associated with change in clinical practice and subsequent health effects included direct and indirect health care costs such as use of x-rays, over-the-counter or prescription analgesics, allied health or GP consults, and the time of volunteer or paid carers. Practice change and subsequent health effects are also expected to impact on direct and indirect costs outside the health sector including waiting time and travel time to attend treatment, productivity gains due to a change in specific disability and time lost from work associated with treatment. Of these, data was only available for a subset of direct costs (lumbar spine and pelvis x-ray and CT scan) by provider (rather than by patient). Specifically, data on total Medicare reimbursements for lumbar spine and pelvis x-ray and CT scans, total Medicare reimbursements and total Medicare patients during the 12 month period after delivery of intervention/control were obtained from Medicare Australia. Our cost-analysis therefore excludes certain indirect health care costs associated with between-group variation in health status (including use of over-the-counter or prescription analgesics, allied health or GP consults, and the time of volunteer or paid carers), as well as direct and indirect costs outside the health sector (including waiting time and travel time to attend treatment, productivity gains due to a change in specific disability, and time lost from work associated with treatment). Health service utilization and cost by group are summarised in Table S5.

**Table S5: Imaging referrals and cost by group, per protocol data**

| **Category** | **Description** | **Number (A)** | **Unit cost (B)** | **Total cost (A x B)** |
| --- | --- | --- | --- | --- |
| **Treatment (GPs=43, Patients=85,899)** | | | | |
| **X-ray** | |  |  |  |
| 57715 - Radiology: Pelvic Girdle | | 196 | $60.90 | $11,936.40 |
| 58106 - Radiology: Lumbosacral | | 419 | $77.00 | $32,263.00 |
| 58108 - Radiology: Four regions | | 6 | $132.90 | $797.40 |
| 58109 - Radiology: Sacrococcygeal | | 28 | $47.00 | $1,316.00 |
| 58112 - Radiology: Two exams | | 146 | $97.25 | $14,198.50 |
| 58115 - Radiology: Three exams | | 36 | $132.90 | $4,784.40 |
| **CT scan** | |  |  |  |
| 56223 - CT: Lumbosacral | | 525 | $240.00 | $126,000.00 |
| 56226 - CT: Lumbosacral with contrast | | 3 | $351.40 | $1,054.20 |
| 56233 - CT: Two exams | | 25 | $240.00 | $6,000.00 |
| 56234 - CT: Two exams with contrast | | 0 | $351.40 | $0.00 |
| 56237 - CT: Three regions | | 0 | $240.00 | $0.00 |
| **PER GROUP** | | **1384** | - | **$198,349.90** |
| **PER GP** | | **32.19** | - | **$4,612.79** |
| **PER PATIENT** | | **0.016** | - | **$2.31** |
| **Control (GPs=47, Patients=82,392)** | | | | |
| **X-ray** | |  |  |  |
| 57715 - Radiology: Pelvic Girdle | | 231 | $60.90 | $14,067.90 |
| 58106 - Radiology: Lumbosacral | | 301 | $77.00 | $23,177.00 |
| 58108 - Radiology: Four regions | | 0 | $132.90 | $0.00 |
| 58109 - Radiology: Sacrococcygeal | | 36 | $47.00 | $1,692.00 |
| 58112 - Radiology: Two exams | | 116 | $97.25 | $11,281.00 |
| 58115 - Radiology: Three exams | | 22 | $132.90 | $2,923.80 |
| **CT scan** | |  |  |  |
| 56223 - CT: Lumbosacral | | 459 | $240.00 | $110,160.00 |
| 56226 - CT: Lumbosacral with contrast | | 3 | $351.40 | $1,054.20 |
| 56233 - CT: Two exams | | 36 | $240.00 | $8,640.00 |
| 56234 - CT: Two exams with contrast | | 2 | $351.40 | $702.80 |
| 56237 - CT: Three regions | | 2 | $240.00 | $480.00 |
| **PER GROUP** | | **1208** | - | **$174,178.70** |
| **PER GP** | | **25.70** | - | **$3,705.93** |
| **PER PATIENT** | | **0.015** | - | **$2.11** |
