# Supplementary material for: Economic Evaluation of Active Implementation versus Guideline Dissemination for Evidence-Based Care of Acute Low-Back Pain in a General Practice Setting
Source: PLoS One. 2013 Oct 11;8(10):e75647. doi: 10.1371/journal.pone.0075647 (PMC3795707; doi:10.1371/journal.pone.0075647)
Supplement: Appendix S6 — Adjustment for differential timing of costs and effects. (DOCX) [file pone.0075647.s006.docx]

**Appendix S6: Adjustment for differential timing of costs and effects**

All costs were expressed in January 2009 AUD by attaching 2009 unit costs where available or inflating actual costs to March quarter 2009 using the all-items Consumer Price Index from the Australian Bureau of Statistics. As trialled, costs associated with development of the IMPLEMENT intervention were incurred up to 12 months prior to commencement of delivery of the intervention/control and up to 16 months prior to commencement of 12 month Medicare Australia data collection period. Where development has already taken place, policy-makers faced with the decision of whether or not to proceed to delivery of the active IMPLEMENT intervention will be concerned only with the trade-off between changes in practice and the costs of delivery. For the within-trial analysis presented here, we make the simplifying assumption that within-trial changes in practice arise on average within the same 12 month period as the costs of delivery of the intervention/control. To inform the decision of whether or not to proceed with delivery of an existing IMPLEMENT intervention, we calculate the ratio of undiscounted incremental costs (excluding development costs) and benefits.

For policy-makers faced with the decision of whether or not to invest in development and subsequent delivery of a similar intervention, the costs associated with development were expended some 12 months prior to receipt of any cost-offsets and/or health gains arising from practice change. Here, we make the simplifying assumption that practice change and costs associated with delivery would occur on average some 12 months subsequent to the initial investment in intervention development. To inform the decision of whether to invest in development and subsequent delivery, we calculate the ratio of discounted incremental costs and benefits as described below.

To inform the decision of whether to invest in development and subsequent delivery, we calculate the present value of total cost (PVC) per GP for treatment/control groups using the following formula:

PVC_Rx_=[(C_Rx_Development_ / (1+r)^0^)+((C_Rx_Delivery_ + C_Rx_Delivery_) / (1+r)^1^)]

PVC_Control_=(C_Control_Delivery_ + C_Control_HSU_) / (1+r)^1^

Where development costs equal zero for the dissemination only control intervention and all cost items are calculated per participating GP. We calculate the present value of outcomes for treatment and control groups using the following formula:

PVO_Rx_=(O_Rx_) / (1+r)^1^

PVO_Control_=(O_Control_) / (1+r)^1^

Where r is equal to the discount rate for both costs and outcomes with r=0.05 in the base-case but 0.03 and 0.07 in sensitivity analysis. C_Rx_Development_, C_Rx_Delivery_ and C_Rx_Delivery_ give the cost of development, delivery and health service utilization in the intervention group. C_Control_Development_, C_Control_Delivery_ and C_Control_Delivery_ give the cost of development, delivery and health service utilization in the control group. O_Rx_  and O_Control_ denote outcomes in treatment and control groups.
